# Optical Surface Management System for Patient Positioning in Interfractional Breast Cancer Radiotherapy

**DOI:** 10.1155/2018/6415497

**Published:** 2018-01-09

**Authors:** Zhao Ma, Wei Zhang, Yi Su, Peiji Liu, Yinghua Pan, Gang Zhang, Yipeng Song

**Affiliations:** ^1^Department of Radiation Oncology, Yantai Yuhuangding Hospital, Yantai 264000, China; ^2^Department of Medical Imaging, Yantai Yuhuangding Hospital, Yantai 264000, China

## Abstract

**Background:**

The Optical Surface Management System (OSMS) is a simple, fast, reproducible, and accurate solution for patient set-up and can minimize random day-to-day set-up errors. However, studies in breast cancer patients are rare.

**Objective:**

To analyze 200 patient set-ups in 20 patients with breast cancer by comparing the OSMS with the conventional cone-beam computed tomography (CBCT).

**Method:**

Displacements from concurrent OSMS and CBCT registrations were compared in a total of 200 setups of 20 patients to analyze the interfractional displacement and positioning displacement in three dimensions (lateral, longitudinal, and vertical directions).

**Results:**

The interfractional displacement on the lateral, longitudinal, and vertical directions for OSMS versus CBCT was 0.049 ± 0.254 versus 0.041 ± 0.244 centimeters (cm); 0.018 ± 0.261 versus 0.040 ± 0.242 cm; 0.062 ± 0.254 versus 0.065 ± 0.240 cm, respectively, without any significant difference (all *P* > 0.05). The duration for CBCT scan was about 60 seconds (s), while that for image processing, matching, and couch displacement was at least 5 minutes (min). The average scanning time with OSMS was less than 20 s, and the total duration for positioning was less than 1 min.

**Conclusion:**

OSMS is an efficient tool to improve the accuracy and increase the speed for verifying the patient positioning in radiotherapy for breast cancer.

## 1. Introduction

Breast cancer is one of the most common malignant tumors in women [1]. The treatment of breast cancer is multidisciplinary and many women need postoperative radiotherapy [2]. Postoperative radiotherapy aims to kill any remaining cancer cells in the breast, hereby improving the local control rate [3].

With the development of new radiotherapy techniques such as intensity modulated radiotherapy (IMRT), volumetric modulated arc therapy (VMAT), and tomotherapy (TOMO), studies have focused on reducing positioning errors in order to improve dose accuracy [4–7]. The reproducible positioning of the patient over the entire course of the radiotherapy is essential for the tumor bed receiving the planned doses of radiation and to decrease toxicity [6, 8]. Indeed, the accuracy of positioning patients with breast cancer during radiotherapy is crucial for its success. More specifically, the patients-positioning accuracy influences not only the actual dose distribution in the target region but also the dose exposure of organs at risk (including lung, heart, and spinal cord). Hence, the improvement of such accuracy can increase the local control rate and decrease the occurrence of complications such as radiation pneumonia and pulmonary fibrosis.

Recently, optical surface imaging has been explored for verifying the patient's pretreatment position and controlling for patient movement during the treatment, achieving agreement of about 1 mm [9–11]. Using this approach, the position of the patient is registered to the planning computed tomography (CT) scan to calculate patient displacement. This approach improves the reproducibility of the patient positioning from one treatment session to the other and allows for treatment interruption if the patients moves [12]. The Optical Surface Management System (OSMS; Varian, Palo Alto, CA, USA) has attractive features for patient setup, monitoring and gating to aid in hitting radiotherapy targets [13]. However, although phantom studies were performed in [9–12], studies in actual patients are rare.

Therefore, the aim of this study is to analyze 200 patient setups in 20 patients with breast cancer by analyzing the reliability and accuracy of OSMS compared with cone-beam CT (CBCT). In the present study, the Catalyst system was used for patient positioning. This system uses three high-power LEDs to project light with wavelengths of 405 (blue), 528 (green), and 624 nm (red) onto the object. The blue component is the measuring light for scanning the object and is detected by a monochrome CCD camera, with an acquisition speed of 202 frames per second. The green and red lights project surface mismatches (actual versus reference scan) onto the area where the mismatch is detected to aid patient positioning. Two custom settings embedded in the Catalyst software, namely, the gain and integration time (IT), can influence scan quality. The gain is the quantity of captured electrons required on a pixel of the CCD camera to convert light into electronic charge and hence a digital readout. IT defines the time of light absorption. The maximum scan volume is 80 cm width, 130 cm length, and 70 cm height. An individual region of interest related to the paradigm can also be defined.

## 2. Materials and Methods

### 2.1. Study Design and Patients

This was a prospective study of 20 patients with breast cancer aged 36–57 years (median, 45 years), who were prescribed to receive radiotherapy at the Department of Radiation Oncology of the Yantai Yuhuangding Hospital between January 2015 and July 2016. The inclusion criteria were (1) breast cancer; (2) being prescribed adjuvant whole breast irradiation (WBI); and (3) receiving 4–8 cycles of chemotherapy before radiotherapy.

Fourteen patients received radiotherapy on the left side and six on the right side. Ten patients had received breast-conserving surgery (all had estrogen receptor-positive tumors and were pT1N0) and 10 had received radical surgery (all had T3-4N0-3 or TxN2-3 disease).

The study was approved by the ethics committee of the Yantai Yuhuangding Hospital. Written informed consent was obtained from each patient.

### 2.2. Accelerator and Position Verification System

**Figure 1:**
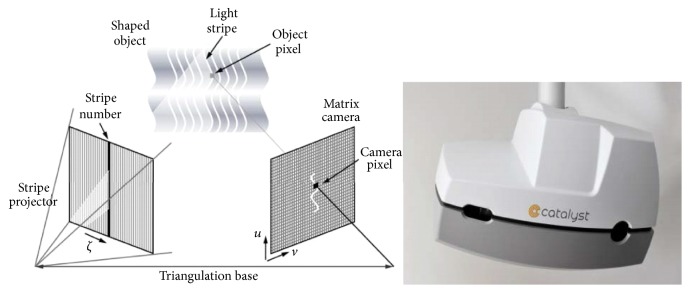
The Catalyst Optical Surface Management System.

A Trilogy medical linear accelerator (Varian, Trilogy, CA, US) is used at our center. The Eclipse system (Varian, Palo Alto, CA, USA) was selected as the treatment planning system. Optical surface scanner with reprojection capabilities (C-RAD Catalyst, Uppsala, Sweden) was used in this study (Figure 1). The OBI system (Varian, Palo Alto, CA, US) was used for CBCT.

### 2.3. CT Reference Image Acquisition and Planning

Patients were placed in an immobilization cradle (WingSTEP™, Elekta Ltd., UK) in the supine position and instructed to breathe normally. All patients received routine training before scanning, and calm breath was needed during the scanning. The patient was positioned with their two arms uplifted, elbows placed on the bracket, and the two hands holding rods. Body films were generally not used for immobilization. Three cross-shaped markers were placed on the body surface and they were positioned according to the surface markers.

An averaged CT (Discovery RT590, GE Healthcare, Waukesha, WI, USA) was performed (reconstructed slice thickness of 5 mm and pitch of 0.15) to account for breathing motions. CT images were obtained from the mandible to 5 cm below the diaphragm, covering the entire chest wall. The CT images were transmitted to the radiotherapy planning system (TPS). Based on the CT information, an automatically generated body outline (larger than −400 Hounsfield units, HU) was contoured in 3D with a point density of the triangulated mesh of about two vertices/cm^2^. This was then used as the CT reference image.

Treatment plans for all 20 patients were concluded with the use of the Eclipse 11.0 software (Varian, Palo Alto, CA, US). Planning target volumes (PTVs) were contoured by the treating physicians (volumes of 524–1425 cc, mean of 864 cc). The contours of the skin, lungs, and bones were sketched automatically by the system. Radiotherapy was performed with 6-MV X-ray using two tangent conformal fields (70–80% of total prescription) and two ARC fields (20–30% of total prescription). The two arcs were in an angle of 60 ± 20° in order to reduce the radiation dose to the ipsilateral lung. The prescription dose to the PTV was 50 Gy (25 fractions of 2 Gy). Patients with breast-conserving surgery had an additional 10 Gy for gross tumor volume (GTV). 98% of the prescription dose distribution covered 95% of the volume, in accordance with the International Commission on Radiation Units 83^#^ report (ICRU83^#^).

### 2.4. OSMS

**Figure 2:**
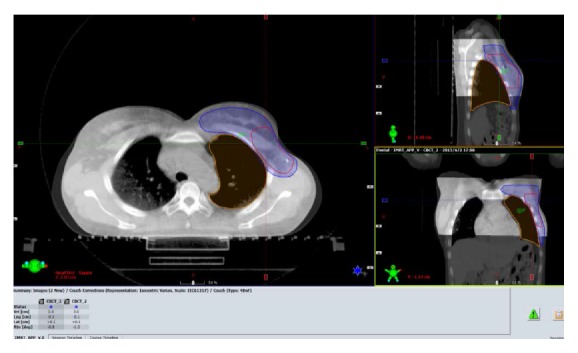
The cone-beam computed tomography (CBCT) registration of the treatment areas. The table below the image shows the displacement after the CBCT registration.

**Figure 3:**
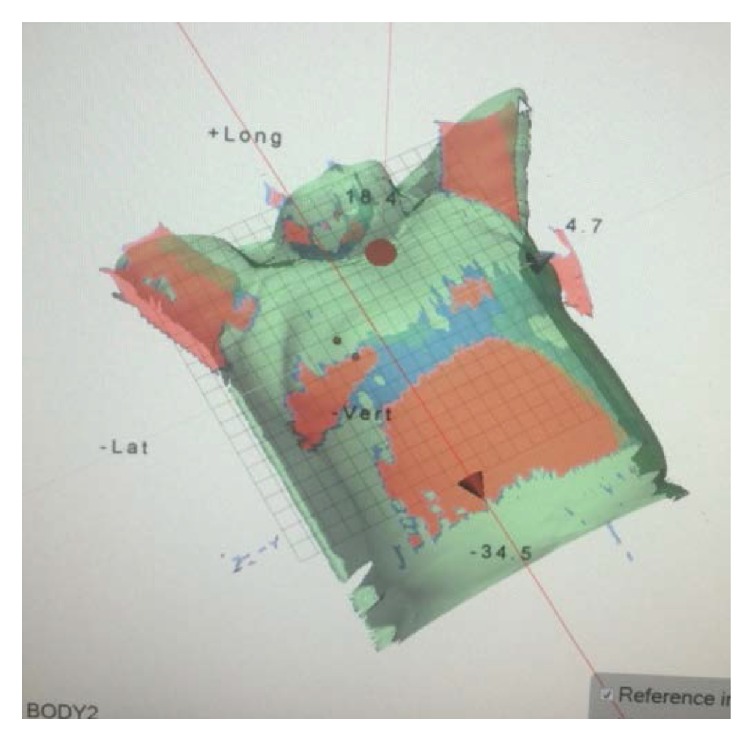
Illustration of collecting the body surface information of the patients using the Optical Surface Management System.

**Figure 4:**
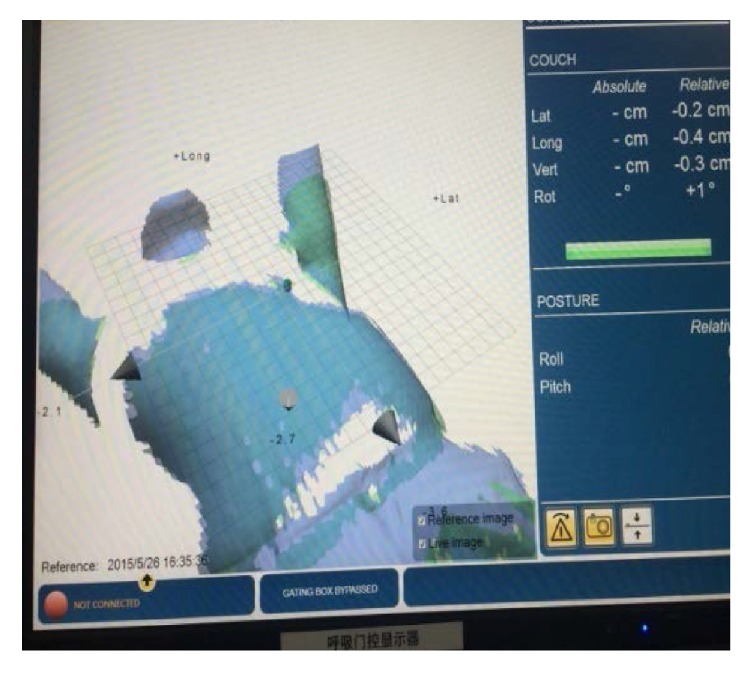
The calculation of the setup errors by the OSMS registration.

**Figure 5:**
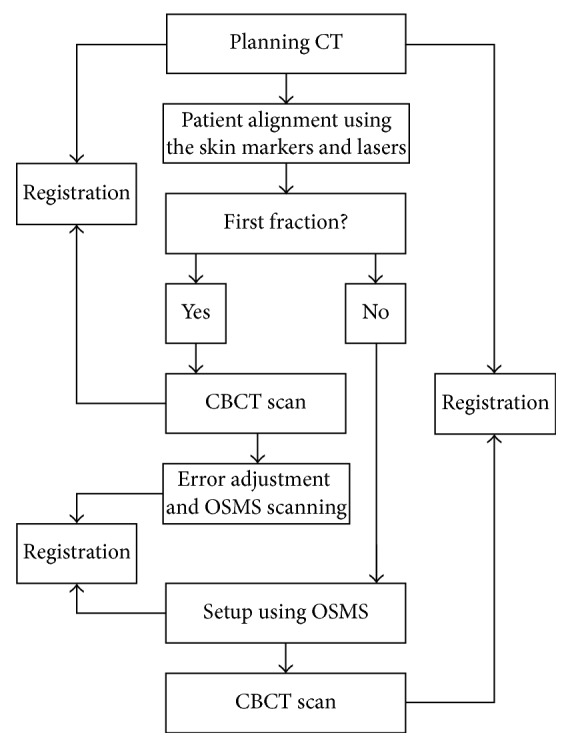
Study flowchart.

The patients were positioned according to the markings on the patients' body surface and were further verified using a CBCT scan prior to the first treatment. Bony structures from the planning CT were used as a reference for the CBCT method. After matching the registration CT reference image (CTref), the displacement was acquired (Figure 2). The position was fixed with the error data generated by the system, and the images of the patient surface were collected by the OSMS as reference images (OSMS_ref_) for the subsequent treatments (Figure 3). For the second treatment, the OSMS_ref_ was used to correct the position. Optical surface images were collected to obtain the displacement in all three directions including LAT, LONG, and VERT, and the errors were required to be less than 5 mm. Then, the position was validated using CBCT to determine the displacements in the three axes. Displacements from concurrent OSMS (Figure 4) and CBCT registrations were compared for the 20 patients for a total of 200 setups.  Figure 5 presents the study flowchart.

### 2.5. Statistical Analysis

Continuous data were presented as mean ± standard deviation and analyzed using the paired *t*-test. Bland-Altman plots were used to determine the agreement between the two methods. Statistical analysis was performed with SPSS 16.0 (IBM, Armonk, NY, US) and MedCalc (MedCalc Software bvba, Ostend, Belgium). Two-sided *P* values were considered statistically significant.

## 3. Results

### 3.1. Interfractional Displacements

**Figure 6:**
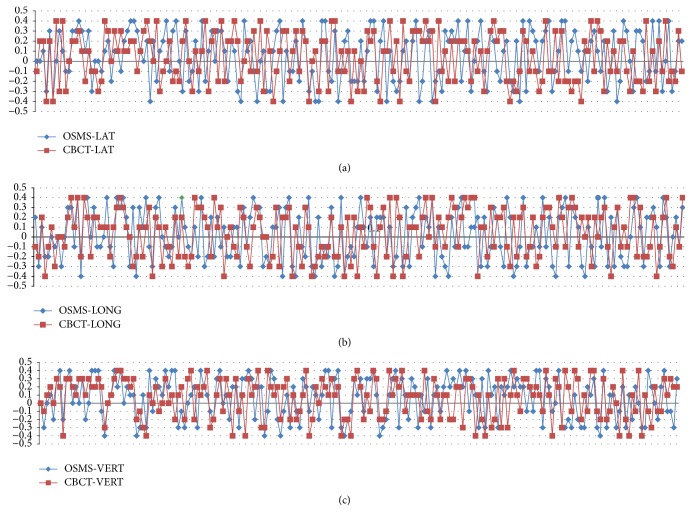
(a) Interfractional displacement (cm) of 200 setups in 20 breast cancer patients in the LAT direction using the OSMS and CBCT scan. The horizontal axis represents the 200 sets of data from the 20 breast cancer patients on the LAT direction, and the vertical axis represents the interfractional displacement between the OSMS and CBCT scan. The blue line shows the displacement of the OSMS scan; the red line indicates the displacement of the CBCT scan. (b) Interfractional displacement (cm) of 200 setups in 20 breast cancer patients in the LONG direction using OSMS and CBCT scan. The horizontal axis represents the 200 sets of data from the 20 breast cancer patients on the LONG direction, and the vertical axis represents the interfractional displacement between the OSMS and CBCT scan. The blue line shows the displacement of the OSMS scan; the red line indicates the displacement of the CBCT scan. (c) Interfractional displacement (cm) of 200 setups in 20 breast cancer patients in the VERT direction using OSMS and CBCT scan. The horizontal axis represents the 200 sets of data from the 20 breast cancer patients on the VERT direction, and the vertical axis represents the interfractional displacement between the OSMS and CBCT scan. The blue line indicates the displacement of the OSMS scan; the red line shows the displacement of the CBCT scan.

**Figure 7:**
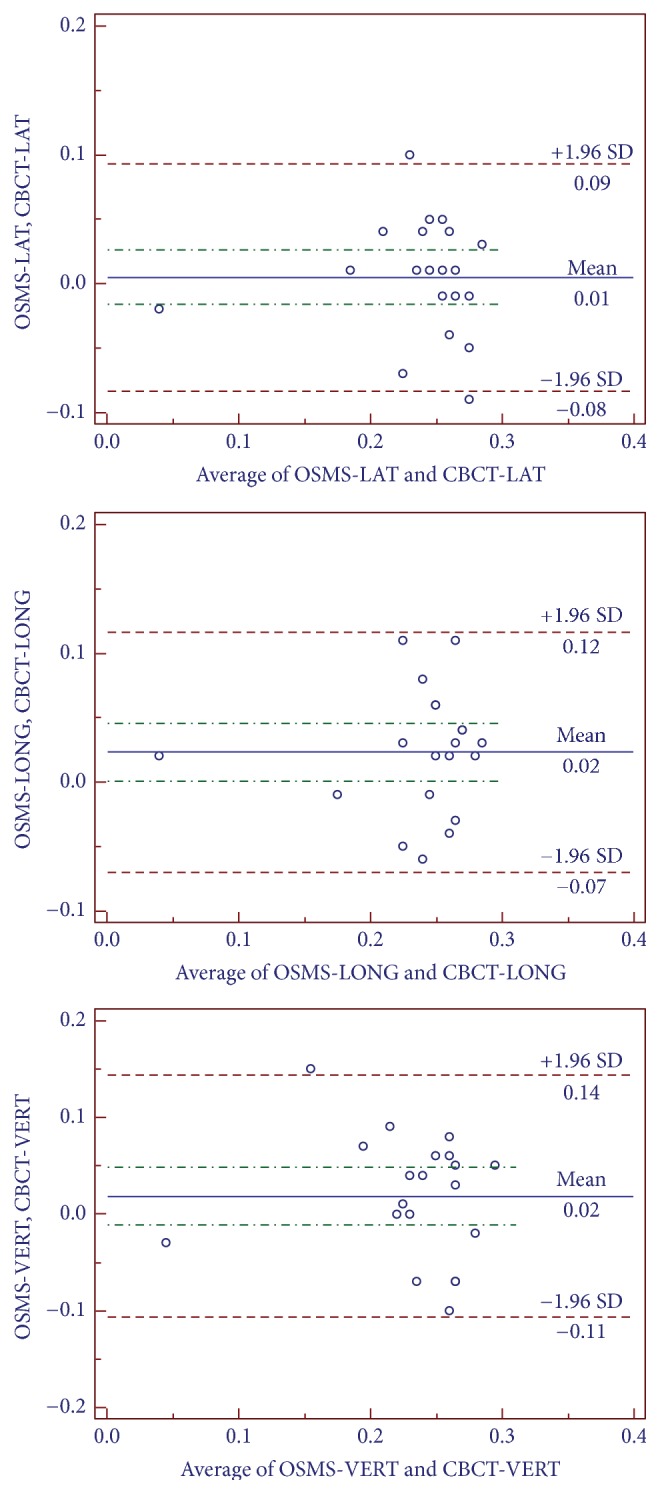
Analysis of Bland-Altman consistency in the three axes. The graphs show the mean value of 10 setups for each of the 20 patients; that is, a total of 200 setups are considered.

For the 200 setups, the interfractional displacements on the LAT, LONG, and VERT directions for OSMS versus CBCT were 0.049 ± 0.254 versus 0.041 ± 0.244 cm; 0.018 ± 0.261 versus 0.040 ± 0.242 cm; 0.062 ± 0.254 versus 0.065 ± 0.240 cm, respectively, without any significant difference (all *P* > 0.05) (Table 1). The total time for setup, registration, and correction was 66.8 ± 17.7 versus 308.0 ± 10.3 s for OSMS and CBCT, respectively. The time for OSMS was significantly shorter than for CBCT.  Figure 6 shows the interfractional displacement in the LAT direction, the displacement in the LONG direction, and the displacement in the VERT direction.  Figure 7 shows the Bland-Altman consistency analysis.

### 3.2. Overall Positioning Errors

Table 2 presents the overall positioning errors for the 20 patients. Similar displacement was observed in OSMS and CBCT scan, without differences.

## 4. Discussion

External beam radiotherapy requires reproducible and precise patient positioning and continuous monitoring. The OSMS shows promising accuracy, but studies in actual breast cancer patients are rare. Therefore, this study aimed to analyze 200 patient setups in 20 patients with breast cancer by comparing OSMS with CBCT. The results showed that OSMS is an efficient tool to improve the accuracy and increase the speed for verifying and complementing patient positioning in radiotherapy for breast cancer.

Systems for target delineation and patient positioning can be divided into radiographic imaging (such as X-ray imaging) and nonradioactive systems. Imaging and positioning systems (e.g., nonradioactive optical scanning systems) can be used to obtain accurate 3D information of patients, based on 2D data input. CBCT is a standard method for verifying the position. With excellent 3D imaging capabilities and high kV-level resolution, this method has become an important means of position verification prior to radiotherapy [13–16]. Hence, CBCT correction should be adopted prior to the treatment for the purpose of preventing the positioning errors. A study by Wang and Li [17] proposed to use the Varian Airborne KV CBCT system for positioning errors of breast cancer patients. In this work, it was shown that the maximum displacements in left-right (LR), anterior-posterior (AP), and superior-inferior (SI) directions were 0.22 cm, 0.49 cm, and 0.48 cm, respectively, before the CBCT correction; and the values became 0.16 cm, 0.21 cm, and 0.17 cm, respectively, after the CBCT correction. Furthermore, based on 758 patients, Wu and Li [18] showed that the average positioning errors in the LR, AP, and SI directions were −0.5 ± 2.8 mm, 0 ± 3.0 mm, and 0.4 ± 3.4 mm, respectively. The corresponding outside boundaries calculated by the formula were 3.2 mm, 2.1 mm, and 3.4 mm, respectively. By contrast, in the present study, errors of CBCT scanning of the 20 breast cancer patients in the 3D direction were all below 0.5 cm, which is similar to the previous studies. In addition, the results showed good agreement between OSMS and CBCT. This suggests that OSMS can be used as an effective measure to increase precision of patient positioning during breast cancer radiotherapy. Note that there are some limitations of CBCT in observing the repeatability of patient positioning every day, monitoring positioning during treatment, the influence of organ movement on treatment precision, and so on. Therefore, the OSMS effectively complements the CBCT as it allows for dynamic monitoring and real-time tracking of the patient's surface position during therapy. The results of the present study are supported by previous studies in breast cancer patients [19] and in other types of targets [13].

Furthermore, note that CBCT requires the use of X-ray, and thus the patients receive additional radiation doses during the treatment. Zhang and Gao [20] showed that if a cylindrical measurement phantom with a diameter of 15 cm and length of 10 cm was used, and further exposed to 6MV X-ray to irradiate 5MU every time, then the single dose was 0.82–1.00 cGy and the total dose was 24.6–30.0 cGy for 30 consecutive scans. For kV-level 2D imaging and using the same phantom to measure with the exposure parameters being 100 kV, 100 mA, and 80 ms, the resulting doses were 0.46–1.04 mGy for a single scan and the total dose was 13.83–30.32 mGy for 30 consecutive scans. Using CBCT imaging, under standard conditions of 100 kV, 20 mA, and 20 ms, the resulting doses were 2.99–6.42 mGy for a single scan and the total dose for 30 scans was 10–20 cGy. This suggests that the extra irradiation can cause some harm to the patients. Hence it is required to strictly limit the number of MV scans to ensure maximum patient safety [16]. The kV imaging dose is less than 1% of the conventional fractionation. Nevertheless, considering different biological effects of kV-level X-ray and MV-level ray, the imaging dose is not subtracted from the treatment plan. The OSMS can project visible light on to the body surface and thus does not increase the patient's radiation dose.

CBCT scanning needs a CBCT gantry rotating to obtain and reconstruct a CT image within volume [21]. Stieler et al. [14] showed that the scanning time with CBCT is about 60 s and that of the optical imaging system is less than 5 s. The present study showed that it took about 5 min for the whole process of CBCT scanning, image reconstruction, and error correction, while the positioning time of the optical image system was no more than 20 s. This reduced the patient position movement caused by his/her poor tolerance and allowed timely monitoring of the patient's change in position without delaying the extra treatment time. It can simplify the patient positioning process and provides a surveillance function to detect patient movement or breathing during treatment.

There are some limitations of OSMS, such as greater errors in imaging for relatively deeper target areas, insensitivity to fluctuations of smooth surface (e.g., the patient with fixed body film on surface), and blind angle at the neck. Stieler et al. [14] showed that the imaging quality of OSMS is influenced by the surface shape and color. A phantom was used to simulate profile changes of the head and neck, pelvic cavity, and breast; the results showed that the vertical surface and high gain setting caused excessive exposure, while horizontal surface needed more integration time and higher gain. Therefore, the system setting must be customized for different target areas with different standards (head and neck/breast/pelvis). An effective verification method does not exist currently for the optical surface system versus deformable registration. Hence, further research and study are required in the future. In order to solve these problems, the positioning error is generally minimized by adjusting tolerance, changing patient supine-prone position, coordinating respiratory gating, and installing three OSMS systems pointing in different directions.

An advantage of OSMS is the real-time monitoring in the entire treatment process. When the patient's breathing rate exceeds a certain threshold (e.g., longer than 1 cm), the radiation beam is shut down to prevent toxicity. In addition to Catalyst, the OSMS also has a Real-time Position Management (RPM) system. The RPM system (Varian Medical System Company, Palo Alto, CA, USA) is aligned to the patient by an infrared source and camera. This device is installed at the foot end of the couch. It is installed on a plastic box with a reflective marker on the breast of a cancer patient to track his/her breathing movement.

Bekke et al. [22] used a manikin to simulate sinusoidal breathing and estimated amplitude, period, and baseline (signal value at end-expiration) with the RPM and Catalyst systems. Compared with the accelerator's guiding lasers, the Catalyst measurements showed better correlations than the RPM system, and larger baseline errors were seen with RPM.

The present study still has some limitations. The sample size was small and all patients were from the same hospital and thus possibly introducing some bias.

In conclusion, the OSMS is an efficient tool to improve the accuracy and speed for verifying and complementing patient positioning in radiotherapy for breast cancer. OSMS could be used in future potential applications in gating, adaptive therapy, and 3D or 4D image fusion between most imaging modalities and image processing [16].

## Figures and Tables

**Table 1:** Interfractional displacements of the 200 setups in 20 patients with breast cancer.

Parameter	LAT (cm)		LONG (cm)		VERT (cm)		Time (s)	
	OSMS	CBCT	OSMS	CBCT	OSMS	CBCT	OSMS	CBCT
Mean	0.049	0.041	0.018	0.040	0.062	0.065	66.810	308.040
Standard deviation	0.254	0.244	0.261	0.242	0.254	0.240	17.732	10.283
*t*-value	0.330		−1.029		−0.126		−215.262	
*P* value	0.742		0.305		0.900		0.000	

**Table 2:** Comparison of overall positioning errors in 20 breast cancer patients using OSMS and CBCT scan.

Patient number	(1)	(2)	(3)	(4)	(5)	(6)	(7)	(8)	(9)	(10)
OSMS (cm)	0.22 ± 0.05	0.25 ± 0.03	0.26 ± 0.03	0.28 ± 0.03	0.25 ± 0.04	0.24 ± 0.01	0.25 ± 0.02	0.29 ± 0.02	0.03 ± 0.01	0.28 ± 0.04
CBCT (cm)	0.24 ± 0.07	0.16 ± 0.07	0.19 ± 0.03	0.23 ± 0.02	0.23 ± 0.02	0.25 ± 0.02	0.24 ± 0.05	0.25 ± 0.02	0.05 ± 0.02	0.27 ± 0.02
*P* value	0.65	0.13	0.15	0.17	0.18	0.18	0.94	0.15	0.58	0.67

Patient number	(11)	(12)	(13)	(14)	(15)	(16)	(17)	(18)	(19)	(20)

OSMS (cm)	0.26 ± 0.05	0.27 ± 0.02	0.26 ± 0.03	0.26 ± 0.05	0.24 ± 0.02	0.26 ± 0.03	0.25 ± 0.03	0.28 ± 0.02	0.25 ± 0.03	0.26 ± 0.02
CBCT (cm)	0.26 ± 0.01	0.24 ± 0.06	0.23 ± 0.04	0.25 ± 0.04	0.26 ± 0.02	0.21 ± 0.04	0.26 ± 0.05	0.24 ± 0.01	0.27 ± 0.03	0.24 ± 0.04
*P* value	1.00	0.38	0.50	0.83	0.65	0.09	0.88	0.11	0.61	0.55

OSMS: Optical Surface Management System; CBCT: cone-beam computed tomography. The *P* value was obtained using paired *t*-tests.
